# Late-Life Suicidal Ideation in Nordic and Central-European Countries: Gender Differences and Risk Factors from the SHARE Study

**DOI:** 10.1007/s10823-026-09576-x

**Published:** 2026-05-07

**Authors:** Kristina Gundersen, Dagmar Dzurova, Ladislav Csemy

**Affiliations:** 1 Department of Social Geography and Regional Development, Faculty of Science, Research Centre on Health, Quality of Life and Lifestyle in a Geodemographic and Socioeconomic Context (GeoQol), Charles University, Prague, Czechia Czechia; 2 Research Centre on Health, Department of Social Geography and Regional Development, Faculty of Science, Quality of Life and Lifestyle in a Geodemographic and Socioeconomic Context (GeoQol), Charles University, Prague, Czechia Czechia; 3 Department of Social Psychiatry, National Institute of Mental Health, Klecany, Czechia

**Keywords:** Late-life suicidal ideation, Logistic regression, Risk factors, SHARE data, European countries, Nordic region, Visegrad region

## Abstract

Older adults are disproportionately affected by suicide, yet suicidal ideation in this population remains underexplored. This study examines gender-specific risk factors and regional differences in late-life suicidal ideation across Nordic and Visegrad countries, with loneliness as a key social determinant. This study analysed data from 11,712 participants aged 50 years and older from seven European countries (Nordic and Visegrad regions), drawing on Wave 8 of the Survey of Health, Ageing and Retirement in Europe (SHARE). Logistic regression was used to examine gender- and region-specific associations with late-life suicidal ideation. Increased loneliness was strongly associated with higher odds of suicidal thoughts in both men and women. Higher education acts as a protective factor, with middle and high education reducing risk in men, and high education reducing risk in women. Living with a partner lowers suicidal thoughts for women but not for men. Age showed no consistent association, and Nordic men show a lower risk compared to their Visegrad counterparts, whereas no regional differences observed for women. Social and contextual factors, especially loneliness, education, and partnership, shape late-life suicidal ideation in gender- and region-specific ways, highlighting the need for targeted preventive interventions.

## Introduction

Late-life mental health has become an increasingly important public health concern as populations age worldwide. Although suicide mortality has received considerable attention in epidemiological research (Pathirathna et al., 2022; Yan et al., 2023), much less emphasis has been placed on suicidal ideation as an earlier and more prevalent expression of psychological distress among older adults (Simon, 1989). Suicidal ideation is clinically and socially significant because it reflects suffering that may not result in death but nonetheless signals vulnerability and elevated risk. Understanding suicidal ideation is therefore essential for prevention efforts that aim to intervene before thoughts of self-harm escalate into attempts or completed suicide.

Suicidality-related cognitions in later life span a continuum from passive death wishes to more active suicidal ideation (Bachert, 2024). This distinction is particularly important in older populations, where death wishes may reflect multifactorial late-life experiences such as functional decline, bereavement, existential concerns, and social disconnection rather than imminent suicidal behaviour (Kjølseth et al., 2010; Crocker et al., 2006). Nevertheless, both passive and active forms of suicidal ideation are clinically meaningful indicators of distress and warrant careful assessment.

From a sociological perspective, suicidal ideation in later life arises from the interaction between individual vulnerability and social context (Hawton & Pirkis, 2017). Prior research has identified loneliness and social isolation as key correlates of suicidal thoughts among older adults (Hawkley et al., 2016; Lee, 2021). At the same time, social inequalities and differential access to health care and support services may influence whether psychological distress develops into suicidal ideation (WHO, 2014). However, the extent to which these social factors operate similarly across different regional contexts remains insufficiently understood.

Comparative research suggests that broader socio-geographical contexts may shape late-life mental health through differences in historical trajectories, welfare institutions, and social norms (WHO, 2014). In this study, we compare older adults in Nordic EU countries and Visegrad countries within the Central European region, known as the Visegrad Group (V4). These countries’ shared historical and political experiences provide a consistent context for analysing suicidal ideation. These two European regions share demographic ageing trends but differ in social and institutional development. Previous studies have documented regional variation in suicide mortality across Europe (Levi et al., 2003; Eurostat, 2020), yet far less is known about whether comparable patterns exist for suicidal ideation. Examining ideation allows for a more sensitive assessment of psychological distress before it culminates in fatal outcomes and offers insight into potentially preventable stages of suicidality.

As far as we know, relatively little research has examined how gender interacts with regional socio-geographical contexts in shaping late-life suicidal ideation. Moreover, the roles of loneliness, social isolation, educational attainment, and partnership status remain underexplored within comparative regional frameworks. Addressing these gaps, the present study investigates how regional context and gender intersect with key social and socioeconomic risk factors to influence suicidal ideation among older adults in Nordic and Visegrad countries.

This paper focuses on late-life suicidal ideation and aims to support the United Nations’ Sustainable Development Goals (SDGs), specifically Target 3.4, which seeks to improve mental health by reducing suicidal behaviour. As the global population ages rapidly, the number of older adults is increasing significantly. By examining gendered and regional patterns of suicidal ideation, this paper contributes to a more nuanced understanding of vulnerability and protection in later life. It also aims to inform prevention strategies that address both individual and contextual risk factors. Our study focuses exclusively on social and socio-geographical determinants of suicidal ideation and does not examine suicide attempts or completed suicides.

## Review of the Literature

From a sociological perspective, suicidal behaviour is shaped not only by individual vulnerability but also by social integration, cultural norms, and structural conditions. Durkheim (1897) conceptualized suicide as a social phenomenon linked to the strength of social bonds, arguing that weak social integration increases the risk of egoistic suicide. In later life, major transitions such as retirement, declining health, and bereavement may weaken social ties and elevate psychological distress. Social isolation and reduced social participation are consistently identified as risk factors for poor mental health outcomes among older adults (Gerst-Emerson & Jayawardhana, 2015).

Although most literature has focused on suicide mortality and attempts, suicidal ideation is increasingly recognized as a critical early indicator of vulnerability. Suicidal ideation ranges from passive death wishes to active considerations of self-harm and is clinically and socially significant because it reflects psychological suffering that may precede suicidal behaviour and offers an opportunity for early intervention (Lee, 2021).

The interpersonal theory of suicide posits that suicidal ideation arises primarily from thwarted belongingness and perceived burdensomeness (Van Orden et al., 2010). The three-step theory further suggests that psychological pain and hopelessness can trigger suicidal thoughts, which may intensify when social connectedness is insufficient (Klonsky & May, 2015). These frameworks highlight the central role of social relationships and perceived social value in shaping suicidal ideation.

Empirical research distinguishes between passive and active suicidal ideation. Passive ideation, characterized by thoughts such as wishing to be dead without planning self-harm, is more prevalent in later life and often reflects low life satisfaction, resignation, or existential distress rather than immediate suicidal intent (Szanto et al., 1996; Van Orden et al., 2010; Wastler et al., 2022). Active ideation involves intention or planning and is associated with higher psychological distress. Both forms co-occur but represent distinct constructs, and passive death wishes can predict subsequent attempts and premature mortality (Baca-Garcia et al., 2011).

Several social and demographic factors are linked to suicidal ideation among older adults. Previously mentioned social isolation and loneliness are among the most consistent predictors, reflecting subjective emotional distress rather than objective living arrangements (Hawkley et al., 2016; McClelland et al., 2020; Modglin, 2023). Health-related adversities, including poor physical health, functional limitations, and chronic illness, are strongly associated with passive ideation (Lee, 2021). Material deprivation, lower educational attainment, and cognitive decline may further contribute to feelings of burdensomeness and hopelessness (Kjølseth, 2014).

Although depression is closely related to suicidal ideation, it does not fully account for it. Many diagnostic tools for depression include suicidal ideation as a symptom, which can obscure conceptual distinctions (Lee, 2021). Evidence shows that ideation can occur independently of clinical depression: not all older adults reporting suicidal thoughts meet diagnostic criteria for depression, and a substantial proportion of psychologically distressed adults do not report ideation (Van Orden et al., 2010). This suggests that suicidal ideation constitutes a distinct dimension of late-life distress and warrants targeted assessment.

Gender and partnership status further shape patterns of ideation. Women more frequently report passive suicidal ideation, whereas men show higher suicide mortality (Shaw et al., 2021; Beghi et al., 2021). Widowhood and divorce are associated with elevated suicidal thoughts, particularly among men, due to the loss of emotional support and increased social isolation (Levy et al., 2011).

Despite growing research on ideation, relatively little attention has been paid to socio-geographical variation and the intersection of gender with regional differences. Most comparative studies still focus on mortality rather than earlier, preventable stages of suicidality. This study addresses this gap by examining passive suicidal ideation among older adults and how gender and regional context intersect with social and socioeconomic risk factors in Nordic and Visegrad countries. Building on this gap, the aim of the study was to investigate gender-specific risk factors and regional differences in suicidal ideation across Nordic and Visegrad countries, focusing on loneliness as a key social determinant in later life.

## Data and Methods

For this study, we mainly used data from the Survey of Health, Ageing and Retirement in Europe (SHARE), wave 8 (2022), which was the most recent wave available at the start of our research. In addition, demographic variables were drawn from respondents’ first interview (the baseline interview from previous waves 1–7).

### Study Design and Sample Framework

The analytical sample consists of individuals aged 50 and above from seven European countries. These countries were selected to represent the Nordic region (Denmark, Finland, and Sweden) and the Visegrad region (Czechia, Hungary, Poland, and Slovakia). Although broader interpretations sometimes describe the Visegrad region as comprising five Central European countries, this study deliberately focuses on these four core states to ensure data consistency within the SHARE project data. Norway and Iceland, although part of the Nordic region, are not included because they are not members of the European Union and therefore do not participate in SHARE.

**Fig. 1:**
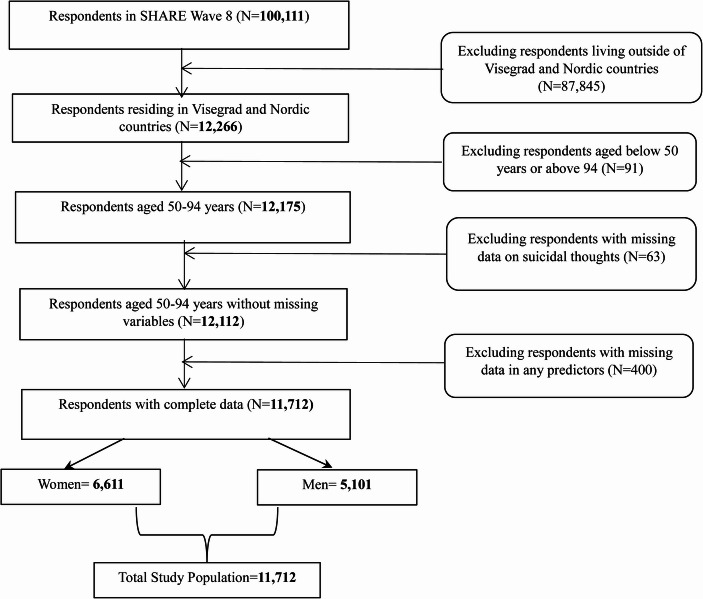
Steps of the sample selection and refinement process

The original SHARE data sample (wave 8) comprised 100,111 observations from Visegrad, Nordic, and other countries (Fig. 1). Respondents residing outside the Nordic and Visegrad regions were excluded, leaving 12,266 observations. The sample was then restricted to individuals aged 50–94 years. Participants younger than 50 and a small number of individuals older than 94 (*N* = 91 in total, of whom 33 were aged above 94) were excluded. The upper age cut-off was applied because the “oldest-old” constitute a very small fraction of the sample in several country × gender strata, which can lead to unstable estimates and problems of quasi-complete separation in logistic regression models. This restriction was therefore adopted to improve the robustness and comparability of estimates across countries and genders. Respondents with more than 25 years of education and those with missing education data were also excluded due to implausible or unverifiable values (*N* = 219). This resulted in a sample of 11,956 observations. Respondents with missing information on suicidal ideation were subsequently excluded, leaving 11,775 observations. Finally, individuals with missing data on loneliness were removed, yielding a final analytical sample of 11,712 respondents: 5,101 men (43.6%) and 6,611 women (56.4%).

#### Dependent Variable: Passive Suicidal Ideation (Binary)

The outcome of this study was suicidal ideation. Suicidal ideation is measured using the SHARE item: “In the last month, have you felt that you would rather be dead?” This item captures passive death ideation rather than active suicidal intent or planning. Accordingly, throughout the manuscript we interpret the dependent variable as passive suicidal ideation and avoid claims about suicidal behaviour that are not measured in the survey. Responses are originally coded as − 2 (“refusal”), − 1 (“don’t know”), 1 (“any mention of suicidal feelings or a wish to be dead”), and 2 (“no such feelings”). For the purposes of the present analysis, we focus on responses coded as 1, the dependent variable is dichotomized, with responses coded as 1 classified as presence of passive suicidal ideation and all other valid responses classified as absence of passive suicidal ideation. This operationalization is consistent with prior research using SHARE data (Lee, 2021).

#### Independent Variables

The study includes basic demographic characteristics, such as age, sex, education level and country of residence and partner in household. Feelings of loneliness are included as a key explanatory variable because subjective social disconnection is a well-established risk factor for late-life suicidal ideation beyond demographic characteristics.

Loneliness is measured using the Three-Item Loneliness Scale (Mehrbrodt et al., 2019), which assesses feelings of lacking companionship, being left out, and feeling isolated. Responses are recorded on a three-point Likert scale (“often,” “some of the time,” “hardly ever or never”), yielding scores from 3 to 9. Scores were categorized as Not lonely (3), Somewhat lonely (4–6), and Very lonely (7–9).

Respondents were grouped into three age categories: 50–64, 65–80, and 81–94 years. These categories reflect distinct stages of later life. The youngest group comprises individuals approaching or entering retirement; the middle group represents early later life characterized by increasing health and social changes; and the oldest group faces advanced aging associated with elevated risks of physical decline, dependency, and bereavement. These categories allow for the identification of age-specific risk patterns.

Years of education are harmonized in SHARE and capped at 25, with values above this threshold recoded as non-substantive responses (“don’t know,” “refusal,” or “suspected wrong”). Consequently, it is not possible to distinguish respondents with more than 25 years of education from those with missing or invalid information. Participants were classified into three educational groups: low (0–10 years), middle (10–14 years), and high (15–25 years). These categories capture meaningful differences in socioeconomic position and cognitive resources relevant to mental health outcomes.

Partnership status was recoded as a binary variable indicating whether the respondent lived with a partner in the household (yes/no).

### Statistical Methods

The analysis provides descriptive statistics and logistic regression models stratified by gender.

We conducted binary logistic regression analyses predicting passive suicidal ideation, with independent variables including feelings of loneliness, age, education level, region, and presence of a partner in the household. Analyses were stratified by gender and based on complete cases for all variables. We estimated three sets of logistic regression models to improve interpretability and to assess robustness. Model 0 reports unadjusted associations by fitting separate single-predictor models; this provides a baseline description of each predictor’s relationship with suicidal ideation and facilitates comparison with descriptive statistics. Model 1 is our main multivariable specification and includes all predictors simultaneously, modelling age as a continuous variable to retain information and minimize over-parameterization. Model 2 repeats the multivariable specification but replaces continuous age with age categories as a sensitivity analysis to assess whether conclusions depend on the assumed linear effect of age and to provide a clinically interpretable age-group comparison. Both modelling strategies were applied to assess the robustness of the results to alternative specifications of age and to evaluate whether potential non-linear or threshold effects of age were masked when age was treated as a continuous variable.

Model fit and diagnostics were assessed using complementary measures to capture different aspects of model performance. Likelihood-ratio tests were used to compare each model with an intercept-only baseline, and discriminative performance was evaluated using the area under the receiver operating characteristic curve (AUC). Explanatory power was assessed with Nagelkerke’s R², and model parsimony was examined using information criteria (AIC/BIC). Calibration was checked using Hosmer–Lemeshow tests.

Results are presented separately for men and women to capture gender-specific associations. All tests were two-sided and statistical significance was set at *p* < 0.05. We report odds ratios (OR) with 95% confidence intervals (CI).

Analyses were conducted using IBM SPSS Statistics 28.0.

## Results

Table 1 shows the distribution of the analytical sample according to the six selected characteristics, stratified by gender. The overall share of people with suicidal ideation was 3.04% for males and 5.34% for females. The highest prevalence of passive suicidal ideation was observed among individuals reporting very lonely feelings of loneliness, with 15.7% of men and 25.1% of women affected. In terms of country of residence, the prevalence of passive suicidal ideation was higher in the Visegrad region, particularly in Poland (men 4.7% and women 7.5%) and Czechia (men 4.4% and women 6.7%). The prevalence of passive suicidal ideation increased with age and decreased with higher levels of education. Individuals without a partner in the household exhibited a higher prevalence of passive suicidal ideation, particularly among women.

**Table 1:** Descriptive statistics of the analytic sample (*N* = 11,712), stratified by gender

Variable	Category	*N*	% within males	Prevalence rate of suicidal thoughts
Male				
Feelings of loneliness	Not lonely	2,997	58.8%	1.2%
	Somewhat lonely	1,850	36.3%	4.2%
	Very lonely	254	5.0%	15.7%
Country of residence	Czechia	998	19.6%	4.4%
	Denmark	962	18.9%	1.2%
	Finland	529	10.4%	2.5%
	Hungary	291	5.7%	3.8%
	Poland	879	17.2%	4.7%
	Slovakia	433	8.5%	1.2%
	Sweden	1,009	19.8%	2.9%
Region	Visegrad	2,601	51.0%	3.9%
	Nordic	2,500	49.0%	2.2%
Age group (in years)	50–64	1,472	28.9%	2.3%
	65–80	2,977	58.4%	3.1%
	81–94	652	12.8%	4.4%
Educational group	Low	1,254	24.6%	5.1%
	Middle	2,585	50.7%	2.6%
	High	1,262	24.7%	1.9%
Partner in household	No	1,015	19.9%	5.1%
	Yes	4,086	80.1%	2.5%
TOTAL		5,101	100.0%	3.04%
Male				
Feelings of loneliness	Not lonely	3,546	53.6%	2.5%
	Somewhat lonely	2,662	40.3%	6.1%
	Very lonely	403	6.1%	25.1%
Country of residence	Czechia	1,588	24.0%	6.7%
	Denmark	1,133	17.1%	3.6%
	Finland	608	9.2%	3.3%
	Hungary	457	6.9%	6.3%
	Poland	1,112	16.8%	7.5%
	Slovakia	536	8.1%	2.6%
	Sweden	1,177	17.8%	5.1%
Region	Visegrad	3,693	55.9%	6.3%
	Nordic	2,918	44.1%	4.1%
Age group (in years)	50–64	2,067	31.3%	4.0%
	65–80	3,748	56.7%	5.1%
	81–94	796	12.0%	9.9%
Educational group	Low	1,826	27.6%	7.9%
	Middle	3,249	49.1%	5.0%
	High	1,536	23.2%	3.1%
Partner in household	No	2,471	37.4%	8.3%
	Yes	4,140	62.6%	3.6%
TOTAL		6,611	100.0%	5.34%

Table 2 presents the odds ratios (OR) for passive suicidal ideation associated with the variables of interest.

**Table 2:** Binary logistic regression analysis predicting suicidal ideation, stratified by gender

Male or female	Independent Variable	Category	Model 0			Model 1			Model 2		
			Odds Ratio	95% CI		Odds Ratio	95% CI		Odds Ratio	95% CI	
Male	Educational groups	(Reference: Low)									
		Middle	0.50	0.35	0.70	0.52	0.35	0.75	0.49	0.33	0.71
		High	0.36	0.22	0.58	0.44	0.27	0.73	0.43	0.26	0.70
	Partner in household	(Reference: No)									
		Yes	0.48	0.34	0.67	0.84	0.58	1.22	0.82	0.57	1.18
	Region	(Reference: Visegrad)									
		Nordic	0.55	0.39	0.76	0.62	0.43	0.89	0.63	0.44	0.90
	Feelings of loneliness	(Reference: Not lonely)									
		Somewhat lonely	3.52	2.37	5.23	3.15	2.10	4.72	3.21	2.14	4.81
		Very lonely	14.95	9.36	23.88	11.58	7.04	19.04	11.99	7.29	19.72
	Age (a continuous variable)		1.04	1.02	1.06	1.02	1.00	1.04			
	Age groups (in years)	(Reference: 50–64)									
		65–80	0.51	0.31	0.84				1.23	0.81	1.86
		81–94	0.69	0.45	1.05				1.24	0.72	2.12
Female	Educational groups	(Reference: Low)									
		Middle	0.61	0.48	0.77	0.81	0.63	1.04	0.79	0.61	1.01
		High	0.36	0.22	0.58	0.62	0.43	0.88	0.60	0.42	0.85
	Partner in household	(Reference: No)									
		Yes	0.40	0.33	0.50	0.66	0.52	0.84	0.64	0.50	0.82
	Region	(Reference: Visegrad)									
		Nordic	0.65	0.52	0.81	0.89	0.70	1.14	0.91	0.71	1.16
	Feelings of loneliness	(Reference: Not lonely)									
		Somewhat lonely	2.49	1.91	3.24	2.11	1.61	2.76	2.12	1.62	2.78
		Very lonely	12.84	9.44	17.47	9.66	6.99	13.36	9.66	6.98	13.36
	Age (a continuous variable)		1.04	1.03	1.05	1.02	1.00	1.03			
	Age groups (in years)	(Reference: 50–64)									
		65–80	0.38	0.27	0.52				1.10	0.83	1.46
		81–94	0.49	0.37	0.65				1.49	1.03	2.14

ORs are reported with 95% confidence intervals (Cis) and are considered statistically significant at *p* < 0.05 (two-sided), corresponding to 95% CIs that do not include 1. Values of OR > 1 indicates higher odds of suicidal ideation, whereas OR < 1 indicates lower odds

- Model 0: Unadjusted associations.

Single-predictor models show the baseline associations of each independent variable with passive suicidal ideation. Loneliness, lower education, absence of a partner, and residence in Visegrad countries are associated with higher odds of suicidal thoughts in both genders.

- Model 1: Age as a continuous variable.

In the multivariable model including age in years, loneliness is the strongest predictor of passive suicidal ideation for both men (very lonely OR = 11.58, 95% CI 7.04–19.04 for “Very lonely”) and women (OR = 9.66, 95% CI 6.99–13.36). Higher education is protective in both genders, with stronger effects in men (OR = 0.52–0.44) than in women (OR = 0.62, 95% CI 0.43–0.88). Living with a partner significantly reduces the odds of suicidal ideation in women (OR = 0.66, 95% CI 0.52–0.84), but not in men. Men residing in Nordic countries show lower odds compared to those in Visegrad countries (OR = 0.62, 95% CI 0.43–0.89), whereas regional differences are not significant for women. Nagelkerke’s R² indicates modest explanatory power (≈ 0.12).

- Model 2: Age grouped.

Results are consistent when age is categorized (50–64, 65–80, 81–94 years). Loneliness remains the strongest predictor, education remains protective, and living with a partner is protective for women. Age groups generally do not show significant associations with suicidal ideation; an exception is observed for women aged 81–94, who show higher odds after controlling for social and regional factors. Regional effects remain significant for men but not for women. Nagelkerke’s R² is like Model 1 (≈ 0.12).

Categorizing age does not materially change the overall model structure or explanatory power but reveals a modest age-specific effect among women aged 81–94 years that is not captured when age is modelled continuously.

### Summary

Across models and genders, higher loneliness markedly increases the odds of passive suicidal ideation, while higher education is protective, particularly for men. Living with a partner reduces the odds for women. Age, when categorized, generally does not significantly affect suicidal ideation, although women aged 81–94 show higher odds in the fully adjusted model after controlling for social and regional factors. Regional differences are more pronounced in men, with lower risk in Nordic countries compared to Visegrad countries, whereas regional effects are not significant for women.

Table 3 summarizes model fit and diagnostic statistics for the multivariable logistic regressions. In both genders, the multivariable models fit the data significantly better than the intercept-only model, as indicated by the likelihood-ratio tests (all *p* < 0.001). Discriminative performance was acceptable: AUC ≈ 0.77 for males, and 0.74 for females, whereas explanatory power was modest (Nagelkerke’s R² ≈ 0.12). Overall fit was similar for models with continuous versus categorical age, and Hosmer–Lemeshow tests indicated adequate calibration (all *p* > 0.05).

The modest explanatory power of the models suggests that late-life suicidal ideation is influenced by additional unobserved factors, including health status, life-course experiences, and psychosocial vulnerabilities. Together, these findings underscore the importance of early, socially informed interventions targeting loneliness, relationship dynamics, and educational disparities to reduce suicidal thoughts and promote mental health among older adults.

**Table 3:** Model fit and diagnostics

Gender	Model	*N*	Events (SI = 1)	LR χ² (df), *p*	AIC	BIC	McFadden *R*²	Nagelkerke *R*²	AUC (ROC)	Hosmer–Lemeshow χ² (df), *p*
Male	Model 1	5,101	155	151.97 (7), *p* < 0.001	1252.34	1304.64	0.109	0.123	0.771	6.53 (8), *p* = 0.588
Male	Model 2	5,101	155	148.89 (8), *p* < 0.001	1257.42	1316.26	0.107	0.121	0.767	11.96 (8), *p* = 0.153
Female	Model 1	6,611	353	286.22 (7), *p* < 0.001	2485.18	2539.55	0.104	0.124	0.740	7.88 (8), *p* = 0.445
Female	Model 2	6,611	353	284.73 (8), *p* < 0.001	2488.67	2549.84	0.103	0.124	0.740	14.52 (8), *p* = 0.069

## Discussion

This study aimed to examine gender-specific risk factors and regional differences in suicidal ideation across Nordic and Visegrad countries, with loneliness as a key characteristic of social determinants in later life. Our findings indicate that suicidal ideation among older adults is strongly shaped by social and contextual factors rather than by age alone. Loneliness emerged as the strongest and most consistent predictor of suicidal thoughts for both men and women, while education and partnership status showed gender-specific effects. Regional context was also relevant, as men living in Visegrad countries exhibited higher odds of suicidal ideation than their counterparts in Nordic countries, whereas no significant regional differences were observed among women.

These results are broadly consistent with previous SHARE-based research on passive suicidal ideation. Using SHARE Wave 7, Lee (2021) reported substantial cross-national variation in suicidal thoughts and consistently higher prevalence among women and identified loneliness and poor health as key correlates of passive death wishes in later life. By using SHARE Wave 8 data and adopting a different analytical approach that emphasizes socio-geographical regions and gender interactions, the present study extends this work by showing that regional context operates differently for men and women and that social determinants remain central to understanding suicidal ideation in later life. Together, these findings reinforce the importance of treating suicidal ideation as a distinct outcome from suicide mortality and as a critical target for early intervention and prevention (Klonsky & May, 2015; Snowdon, 2018; Schouler-Ocak & Khan, 2024).

Previous research suggests that gender differences in loneliness are often small and inconsistent once confounding factors are considered, although women in later life may experience more intense loneliness due to higher rates of widowhood and longer life expectancy (Pagan & Malo, 2024). These patterns provide an important background for interpreting gendered experiences of loneliness in older populations.

The regression analyses in our study demonstrate a strong and consistent association between loneliness and suicidal thoughts for both men and women. As loneliness increases, the odds of reporting suicidal thoughts rise markedly in both subsamples. This finding is in line with a substantial body of literature identifying loneliness as a key risk factor for suicidal ideation across genders (Cacioppo et al., 2006). Importantly, the SHARE item used in this study primarily captures passive death wishes rather than active suicidal intent, as documented in previous SHARE-based research (Lee, 2021). Loneliness may therefore be particularly influential in shaping passive suicidal ideation, consistent with theoretical models of late-life suicidal risk that emphasise social disconnection, hopelessness, and perceived burdensomeness (Schouler-Ocak & Khan, 2024). In this sense, our findings also align with Durkheim’s classic argument that weakened social integration increases vulnerability to suicidal outcomes.

Education emerges as a significant protective factor against suicidal thoughts, with notable differences between genders. For men, both middle and high education levels significantly reduce the likelihood of suicidal thoughts, whereas for women, only a high education level shows a significant protective effect in our sample. These gender differences may reflect varying societal roles and expectations, where higher education potentially provides men with greater socioeconomic stability and resilience against stressors (Stack, 2000). For women, the benefits of higher education may also relate to increased social support networks and better access to mental health resources (Cutler & Lleras-Muney, 2010). The nuanced impact of education on suicidal thoughts highlights the need to consider gender- specific pathways when examining protective factors in later life.

Previous research indicates that living alone, especially for widows and widowers, increases the risk of loneliness, which in turn is associated with suicidal thoughts (Pagan & Malo, 2024). Our results show that living with a partner significantly decreases the likelihood of experiencing suicidal thoughts for women, but not for men. This gender-specific effect suggests that women may derive greater emotional and social support from cohabiting relationships, which buffers against suicidal ideation. In contrast, men may not experience the same protective effect, possibly due to traditional gender norms emphasizing self-reliance and emotional stoicism (Canetto & Sakinofsky, 2010). Some studies further indicate that risk factors for suicidal thoughts may operate differently across socio-demographic groups and social contexts (Fung & Chan, 2011). Additionally, the duration of the suicidal process tends to be shorter in men than in women, while the higher lethality of suicide methods among men may explain observed gender differences in completed suicides (Schrijvers et al., 2012; De Leo & Kolves, 2017), without contradicting patterns in suicidal thoughts.

Although our study focuses broadly on suicidal thoughts, evidence indicates that older adults may experience mainly passive ideation, while younger older adults may more often report active suicidal thoughts (Cabello et al., 2021; Wastler et al., 2022). This distinction supports including participants aged 50–64 in the “late-life” sample, as it allows capturing age-related differences in the prevalence and nature of suicidal ideation.

Contrary to expectations, our results show no significant differences in suicidal thoughts across age groups for either gender. This indicates that chronological age, within the studied range, does not independently influence the likelihood of suicidal thoughts. Instead, other factors such as loneliness, education, and partnership status appear to play more critical roles in shaping suicidal ideation in later life (Waern et al., 2003).

A notable pattern concerns the oldest female age group (81–94 years), whose association with suicidal ideation changes across model specifications. In the unadjusted model, women in this age group show different odds of suicidal ideation compared with those aged 50–64 years; however, once loneliness, partnership status, education, and regional context are included, this association becomes non-significant and shifts in direction. This pattern is consistent with confounding and suppression effects. Older women in this sample are more likely to possess characteristics that are protective against suicidal ideation, such as lower reported loneliness or higher likelihood of co-residing with a partner in some contexts, which may mask vulnerabilities associated with advanced age in crude models. After adjustment, the residual association of age may reflect age-specific risks such as frailty, bereavement, declining health, and reduced autonomy that are not fully captured by the included covariates (De Leo & Giannotti, 2021). Given the cross-sectional design, this pattern should be interpreted as a composition effect rather than evidence of a causal role of age itself.

Although chronological age did not independently predict suicidal thoughts in our sample, the same age may correspond to different health and functional profiles across countries. Such cross-national differences in life expectancy and functional health may influence the interpretation of age effects and regional patterns of suicidal ideation (see Appendix 2 for detailed indicators of life expectancy and Healthy Life Years at birth by country and sex).

These regional differences are consistent with prior evidence showing substantial cross-national variation in Healthy Life Years (HLY) and morbidity compression in Europe (Straka et al., 2024). Although we restrict the sample to respondents aged 50–94 years and adjust for age in the regression models, the same chronological age may correspond to different average health profiles across countries. Life expectancy and HLY differ substantially between the Nordic and Visegrad regions, implying that, for instance, a 75-year-old in Sweden may, on average, be healthier than a 75-year-old in Hungary. Such cross-national differences in health may confound the interpretation of regional effects if unobserved health status is correlated with both country context and suicidal ideation; therefore, we interpret regional contrasts cautiously and treat cross-country heterogeneity in health and functional aging as a limitation (Appendix 2).

As an additional robustness consideration, cross-country differences in population health could be proxied by country-level indicators such as Eurostat HLY or life expectancy. However, given the small number of countries (*N* = 7), we refrain from over-interpreting macro-level adjustments and instead report them, if included, as sensitivity checks.

Comparative research indicates that Nordic and Central–Eastern European countries can be understood as distinct but analytically meaningful regional groupings shaped by different historical and institutional trajectories. Hilmarsson (2021) conceptualizes these countries as small-state clusters within Europe, emphasizing how variations in political economy, welfare arrangements, and EU integration have produced divergent development paths. Research on post-socialist welfare transformations further shows that Central European countries, including the Visegrad group, have followed reform trajectories that differ substantially from those of Nordic welfare states (Szikra, 2014; Borevi, 2017).

Evidence from comparative health and welfare research supports the relevance of a welfare regime perspective for understanding population health outcomes. Welfare regime characteristics account for substantial national-level variation in self-perceived health and exposure to social risks across Europe, with Nordic welfare states generally associated with more favourable outcomes than Central and Eastern European regimes (Eikemo et al., 2008; Widding-Havneraas & Pedersen, 2020; Berkowitz et al., 2024). Nordic countries typically feature universalistic welfare systems with high public social spending, extensive primary care, and community-based services for older adults, reflecting long-standing commitments to prevention, early intervention, and social participation (Esping-Andersen, 1990; Vabø & Szebehely, 2012). In contrast, Visegrad countries combine public provision with a stronger role for family support and market mechanisms, exhibiting lower public expenditure on health and social services and more limited development of community-based mental health care (Fenger, 2007; Cerami & Vanhuysse, 2009; Szikra, 2014; WHO, 2025). Across Europe, mental health care integration into primary care varies, and unmet needs remain higher in countries with less developed community-based services (OECD, 2025; WHO, 2025). These structural differences provide a relevant framework for interpreting regional patterns of late-life suicidal ideation.

Nordic and Visegrad countries also differ in economic and cultural contexts that shape late-life mental health outcomes. Nordic countries generally display higher levels of economic security and GDP per capita, while Visegrad countries experienced rapid post-socialist socioeconomic transformations, resulting in greater heterogeneity in material conditions among older adults. Culturally, Nordic societies emphasize individual autonomy supported by public systems, whereas Visegrad countries place more emphasis on family networks and traditional social roles (Pagan & Malo, 2024). These factors may influence experiences of aging, social isolation, and mental health. Despite shared demographic trends, including population aging and rising demands on health and social care, regional institutional and socio-cultural differences are likely to shape suicidal ideation patterns.

Welfare state differences are reflected in social protection, eldercare, and mental health services. Nordic countries feature universal pensions with high replacement rates, broad social transfers, and low old-age poverty (Esping-Andersen, 1990; Eikemo et al., 2008). Eldercare relies primarily on municipally provided home-help and home-nursing services, with needs-based entitlement and low reliance on long-term institutional care (Szebehely & Meagher, 2018). Mental health care for older adults is integrated into primary and municipal services, emphasizing prevention and early intervention through mechanisms such as primary-care screening for depression and coordination between health and social services (OECD, 2025; WHO, 2025). By contrast, Visegrad countries show lower public spending, more stratified benefits, and higher old-age poverty (Fenger, 2007; Cerami & Vanhuysse, 2009). Eldercare relies more heavily on families, with fewer community-based services and patchy psychosocial support; mental health care often depends on hospitals or psychiatric institutions, resulting in higher unmet need (WHO, 2025). These differences in welfare arrangements and service provision provide a plausible explanation for cross-national variation in older adults’ mental health.

Maskileyson et al. (2021) highlight significant variations in depressive symptoms among older adults across different European countries. Older adults in Scandinavian countries, such as Denmark and Sweden, generally report fewer depressive symptoms compared to their counterparts in other European countries.

Our study finds that men living in Nordic countries have a significantly lower risk of experiencing suicidal thoughts compared to those in Visegrad countries. This regional disparity is not observed in women, indicating that regional effects on suicidal thoughts are more pronounced in men. The lower risk in Nordic countries for men might be attributed to comprehensive social welfare systems, better mental health services, and higher societal support prevalent in these countries (Hagerty et al., 2002). In contrast, we presume, the absence of significant regional differences for women suggests that other factors, possibly related to gender equality and social norms, may mitigate the regional effects observed in men.

Beyond welfare-state and service-related explanations, cultural factors may also shape regional differences in suicidal ideation (Snowdon, 2018). Culture influences how psychological distress is perceived and expressed, as well as norms surrounding help-seeking and disclosure. Previous research highlights that variations in suicide patterns across gender and age groups are partly rooted in sociocultural contexts (Schouler-Ocak & Khan, 2024).

Taken together, our findings suggest that regional differences in suicidal thoughts emerge from a complex interplay of institutional, social, and cultural mechanisms, with gender moderating these effects. It is important to note that suicidal ideation represents only one stage in the continuum of suicidal behaviour, and regional differences in ideation may not directly translate into differences in suicide attempts or completed suicides (Snowdon, 2018; Schouler-Ocak & Khan, 2024).

### Limitations

Several limitations should be considered when interpreting our findings. First, this study focuses on suicidal ideation rather than suicide attempts or completed suicides. As ideation represents an earlier stage in the suicidal continuum, the results should be interpreted with caution and may not fully reflect risk for more severe suicidal outcomes.

Second, potential biases inherent in the SHARE data, such as self-reporting and regional specificity, may limit the generalizability of our findings. Additionally, the cross-sectional design further restricts our ability to infer causality and restricts conclusions to associations observed at a single point in time.

Third, the single-item measure of suicidal thoughts used in this study does not distinguish between transient thoughts, passive death wishes, and active suicidal planning.

A further limitation concerns cultural differences in the reporting of suicidal ideation. Attitudes toward suicide and mental illness vary across societies, and stigma or social norms may influence individuals’ willingness to disclose suicidal thoughts in surveys. Consequently, cross-national differences may partly reflect variation in reporting rather than true differences in prevalence (Snowdon, 2018; Schouler-Ocak & Khan, 2024).

Questions related to suicidality may also be subject to stigma-related non-response. We therefore report the number of ‘Refusal’ and ‘Don’t know’ responses to the dependent-variable item mh004_ in the full SHARE Wave 8 sample (Appendix 3). Refusals were rare and showed no pronounced pattern by age, gender, education, or country, suggesting that non-response to the dependent variable is unlikely to be strongly systematic. Nevertheless, this limitation should be considered when interpreting prevalence estimates.

In addition, country-specific sample size in the analytic sample do not reflect national population sizes due to SHARE’s sampling design, country-specific fieldwork constraints, and the complete-case requirements of this study. The gender composition also varies across countries. Therefore, unweighted descriptive estimates should be interpreted as reflecting the analytic sample rather than nationally representative population proportions.

Finally, the scope of the current research restricts a more in-depth exploration of the underlying factors contributing to the findings. Due to constraints, our analysis could not examine potential causal mechanisms or broader contextual influences. Although we compared Nordic and Visegrad countries as regional groups, preliminary country-level analyses suggest heterogeneity in effect sizes, particularly for education and cohabitation. Some country-by-gender strata included few events, limiting precision and precluding definitive country-level comparisons. Despite these limitations, the results provide a solid empirical basis for future research employing longitudinal designs, richer measurement instruments, and more granular contextual data.

## Conclusion

This study highlights the central role of social factors in late-life suicidal ideation. Loneliness emerged as the strongest risk factor for both men and women, while higher education and living with a partner exerted gender-specific protective effects. Men in Visegrad countries faced higher risk than their Nordic counterparts, whereas regional differences were not observed among women. Age had little influence. At the same time, the modest explanatory power of the models suggests that late-life suicidal ideation is also influenced by additional unobserved factors beyond those captured in this study. Despite the above, these findings emphasize the need for early, socially informed interventions targeting loneliness, relationship dynamics, and educational disparities to reduce suicidal thoughts and promote mental health among older adults.

## Data Availability

The data used in this study were obtained from the Survey of Health, Ageing and Retirement in Europe (SHARE) database. SHARE data is publicly available for research purposes, but access requires registration and agreement to the SHARE conditions of use. The SHARE data can be accessed at: http://www.share-project.org.

## Appendix 1

**Table 4:** List of abbreviations

CI	Confidence Interval
EU	European Union
GDP	Gross Domestic Product
HLY	Healthy Life Years
LE	Life Expectancy
OECD	Organisation for Economic Co-operation and Development
OR	Odds Ratio
R-UCLA	The Revised University of California Los Angeles Loneliness Scale
SD	Standard Deviation
SDG	Sustainable Development Goals
SHARE	Survey of Health, Ageing and Retirement in Europe
UN	United Nations
V4	Visegrad Group
WHO	World Health Organization

## Appendix 2

**Table 5:** Life expectancy and Healthy Life Years (HLY) at birth in 2020, by sex (Eurostat)

Country	Region	LE at birth, men	HLY at birth, men	LE at birth, women	HLY at birth, women
Sweden	Nordic	80.6	72.8	84.2	72.7
Denmark	Nordic	79.7	58.1	83.6	57.7
Finland	Nordic	79.2	57.7	84.8	55.9
Czechia	Visegrad	75.3	60.9	81.3	62.5
Poland	Visegrad	72.4	60.3	80.6	64.3
Hungary	Visegrad	72.2	61.6	78.8	63.5
Slovakia	Visegrad	73.5	56.3	80.4	57.1

## Appendix 3

**Table 6:** Non-substantive responses to the dependent-variable item (mh004_) in SHARE Wave 8

DV response category for mh004_	Code (SHARE)	Count
Refusal	−2	93
Don’t know	−1	922
Total non-substantive responses	—	1,015
